# Translating tDCS into the field of obesity: mechanism-driven approaches

**DOI:** 10.3389/fnhum.2013.00512

**Published:** 2013-08-27

**Authors:** Miguel Alonso-Alonso

**Affiliations:** ^1^Berenson-Allen Center for Noninvasive Brain Stimulation, Division of Cognitive Neurology, Beth Israel Deaconess Medical Center, Harvard Medical SchoolBoston, MA, USA; ^2^Center for the Study of Nutrition Medicine, Department of Surgery, Beth Israel Deaconess Medical Center, Harvard Medical SchoolBoston, MA, USA

**Keywords:** transcranial direct current stimulation, obesity, weight loss, inhibitory control, prefrontal cortex

## Abstract

Transcranial direct current stimulation (tDCS) is emerging as a promising technique for neuromodulation in a variety of clinical conditions. Recent neuroimaging studies suggest that modifying the activity of brain circuits involved in eating behavior could provide therapeutic benefits in obesity. One session of tDCS over the dorsolateral prefrontal cortex can induce an acute decrease in food craving, according to three small clinical trials, but the extension of these findings into the field of obesity remains unexplored. Importantly, there has been little/no interaction of our current understanding of tDCS and its mechanisms with obesity-related research. How can we start closing this gap and rationally guide the translation of tDCS into the field of obesity? In this mini-review I summarize some of the challenges and questions ahead, related to basic science and technical aspects, and suggest future directions.

Obesity is an unmet global medical need. Modification of lifestyle behaviors, i.e., limiting food intake and increasing physical activity, remains the cornerstone treatment in the vast majority of cases, but it is often ineffective (Fabricatore and Wadden, 2006). There is need for innovative approaches to facilitate behavioral changes leading to a successful weight loss.

Recent data from obesity neuroimaging studies point to an imbalance in prefrontal and limbic brain circuits that support cognition- and reward-related aspects of eating behavior (Carnell et al., 2012; Brooks et al., 2013; Vainik et al., 2013). Manipulating brain activity could help rebalance these circuits and translate into beneficial behavioral changes. Three small proof-of-concept studies have reported an acute decrease in food craving following one session of transcranial direct current stimulation (tDCS) aimed at enhancing the activity of the dorsolateral prefrontal cortex (Fregni et al., 2008; Goldman et al., 2011; Montenegro et al., 2012). The gap between the effects reported in these studies and the efficacy standards expected for clinical trials related to weight management (FDA, 2007) seems large at this time. *How can we start closing the gap and rationally guide the translation of tDCS into the field of obesity?*

Obesity is a heterogeneous condition that can result from a variety of behavioral and non-behavioral phenotypes, ranging from purely metabolic causes to extreme cases of compulsive overeating. In this scenario, tailored interventions may be more appropriate than a one-size-fits-all approach. However, we do not know yet what subtypes of obesity could benefit from tDCS. A reasonable starting point for exploratory trials can be the use of tDCS to facilitate changes in eating behavior, and a focus on obesity cases that share certain characteristics, e.g., high levels of eating disinhibition or binge eating.

Where in the brain should tDCS be applied in obesity? Candidate targets include brain regions supporting eating behavior at three key levels of integration: homeostasis, reward and cognition. The first challenge is that, except for cognition, these regions are subcortical, e.g., hypothalamus, insula and nucleus accumbens. It is unclear whether conventional or high-definition tDCS approaches can provide adequate reliability, sensitivity and specificity for such deep targets, which can be better reached via deep brain stimulation (DBS; Halpern et al., 2011). tDCS seems to be more suited for cortical targets, specifically lateral and dorsomedial sectors of the prefrontal cortex that contribute to cognitive control. Neuroimaging studies have shown that successful long-term weight loss maintainers have a pattern of increased activation in the lateral prefrontal cortex during satiation or in response to food cues (DelParigi et al., 2007; McCaffery et al., 2009). This activation is stronger than in control (non-obese) subjects, suggesting that lateral prefrontal hyperactivity may be a compensatory mechanism to overcome obesity in these individuals. Future studies should examine in detail the specific prefrontal-related processes that may underlie success in these subjects and, based on this information, design tDCS interventions to induce similar brain patterns in refractory obese subjects. This will likely require multiple sessions and high-intensity stimulation schemes, as long as safety is not compromised.

A mechanistic approach to the use of tDCS in obesity requires both a brain and a cognitive target, if the intention is to enhance cognitive regulation of food intake. An emerging cognitive target in obesity is inhibitory control, a core component of executive functions that supports self-regulatory processes and goal-oriented eating behavior (Appelhans, 2009; Houben, 2011; Yokum et al., 2012; Vainik et al., 2013). Prior studies have mapped inhibitory control capacity (indexed by performance in response inhibition tasks) to a basic set of brain regions that include inferior frontal gyrus, pre-supplementary motor area, and subthalamic nucleus (Chambers et al., 2009). tDCS is well suited to reach this target according to preliminary computational models (Truong et al., 2013) and experimental data (Juan and Muggleton, 2012). Given that tDCS enhances synaptic plasticity processes related to learning (Stagg and Nitsche, 2011), the combination of tDCS with computerized training of inhibitory control is a good strategy. This can narrow down tDCS-induced plasticity effects to the cognitive process and brain circuit being targeted. A recent study supports the feasibility and efficacy of pairing tDCS with inhibitory control training (Ditye et al., 2012) and two preliminary clinical trials are underway in obesity based on this approach (Clinical trials.gov website; study numbers: NCT01632280, NCT01793766).

Most of what has been learned to date about tDCS as a technique can be extended into obesity, but there are largely unexplored factors that could modify the impact of tDCS on the brain, particularly in obese subjects. First, the potential influence of metabolic/physiological state: being in a weight-reduced or weight-stable state as well as prandial status (fasting/fed) are associated with different underlying brain activity (Tataranni et al., 1999; Rosenbaum et al., 2008). This source of variability may change the predicted effect of tDCS on obesity-related brain networks, as initial brain activation state has an important role in determining the behavioral outcome of brain stimulation (state-dependency; Silvanto et al., 2008). Additionally, weight-loss diets may influence tDCS-induced plasticity mechanisms. Intake of high-fat, low-carbohydrate, ketogenic diets (e.g., Atkins) enhances GABA-A-receptor-mediated intracortical inhibition (Cantello et al., 2007)—an outcome that might alter the dynamics of tDCS after-effects (Nitsche et al., 2004).

There are still significant gaps of knowledge in obesity pathophysiology that make decisions on tDCS study design difficult at this point. One of them is regarding the best time to apply tDCS to maximize benefits: *prior, during*, or *after* weight loss. Is it better to strengthen brain circuits in preparation for a subsequent weight loss challenge or, rather, guide brain remodeling as weight loss takes place and/or transitions into a weight maintenance phase? To be able to answer these questions there is need for fundamental research examining the time course of neurocognitive changes throughout weight loss. The contribution of brain regions will likely vary over time.

Aside from behavioral effects, there may be additional advantages for the use of tDCS in obesity via metabolism. Anodal tDCS applied over the motor cortex promotes brain energy consumption and causes systemic glucose uptake (Binkofski et al., 2011). The origin of these changes is uncertain; they could occur through a depletion of energy in the brain, the activation of hypothalamic energy sensing mechanisms, and/or via effects on the neurohormonal stress systems. More research is needed, but these findings are encouraging, because glucose intolerance and diabetes are common complications of obesity.

Last, from a more technical angle, it is necessary to define optimal tDCS parameters in obesity. Prior studies with tDCS in food craving have used empirically determined protocols (typically pad size: 35 cm^2^, intensity: 2 mA, duration: 20 minutes, montage: bilateral dorsolateral prefrontal cortex). Moving forward there is need for computational models to make a rational selection of parameters and guide future refinements of tDCS protocols. As an example, we have recently examined the impact of head fat variability on current density distribution (Truong et al., 2012, 2013).

In conclusion, the translation of tDCS into the field of obesity is still at a very early stage, with many challenges and open question ahead. There is need for foundational studies that generate an adequate knowledge base and principles to guide the development of this emerging field.

## Conflict of interest statement

The authors declare that the research was conducted in the absence of any commercial or financial relationship that could be construed as a potential conflict of interest.
